# Macro-Morphological Traits of Leaves for Urban Tree Selection for Air Pollution Biomonitoring: A Review

**DOI:** 10.3390/bios12100812

**Published:** 2022-09-30

**Authors:** Karen Rodríguez-Santamaría, Carlos Alfonso Zafra-Mejía, Hugo Alexander Rondón-Quintana

**Affiliations:** 1 Grupo de Investigación INDESOS, Facultad del Medio Ambiente y Recursos Naturales, Universidad Distrital Francisco José de Caldas, Carrera 5 Este #15-82, Bogotá DC E-111711, Colombia; 2 Grupo de Investigación en Ingeniería Ambiental—GIIAUD, Facultad del Medio Ambiente y Recursos Naturales, Universidad Distrital Francisco José de Caldas, Carrera 5 Este #15-82, Bogotá DC E-111711, Colombia; 3 Ingeniería Topográfica, Facultad del Medio Ambiente y Recursos Naturales, Universidad Distrital Francisco José de Caldas, Carrera 5 Este #15-82, Bogotá DC E-111711, Colombia

**Keywords:** urban trees, leaf area, air quality, air pollution, leaf morphology, particulate matter

## Abstract

Urban trees provide different ecosystem benefits, such as improving air quality due to the retention of atmospheric particulate matter (PM) on their leaves. The main objective of this paper was to study, through a systematic literature review, the leaf macro-morphological traits (LMTs) most used for the selection of urban trees as air pollution biomonitors. A citation frequency index was used in scientific databases, where the importance associated with each variable was organized by quartiles (Q). The results suggest that the most biomonitored air pollutants by the LMTs of urban trees were PM between 1–100 µm (Q1 = 0.760), followed by O_3_ (Q2 = 0.586), PM_2.5_ (Q2 = 0.504), and PM_10_ (Q3 = 0.423). PM was probably the most effective air pollutant for studying and evaluating urban air quality in the context of tree LMTs. PM_2.5_ was the fraction most used in these studies. The LMTs most used for PM monitoring were leaf area (Q1) and specific leaf area (Q4). These LMTs were frequently used for their easy measurement and quantification. In urban areas, it was suggested that leaf area was directly related to the amount of PM retained on tree leaves. The PM retained on tree leaves was also used to study other f associated urban air pollutants associated (e.g., heavy metals and hydrocarbons).

## 1. Introduction

Trees in urban areas provide different ecosystem benefits such as air quality improvement, oxygen production, carbon sequestration, climate regulation, noise reduction, and ecological enrichment [1]. Trees also contribute to the health and psychological well-being of human communities [2]. In addition, biomonitoring with trees is an economical and reliable alternative to conventional technologies for detecting the presence of urban air pollutants [3]. However, there is the growing problem in which urban tree composition tends towards homogenization because the presence of species is due to human choices that are usually guided by aesthetics. This ignores climate and other ecological factors that influence the performance of these organisms and ecosystems [4,5]. Thus, the selection of urban tree species is rarely associated with air quality (e.g., retention of atmospheric particles), and is usually based on aesthetic, cultural, and ecological factors, or some type of food functionality.

Selection criteria for urban tree species are appropriate unless their great diversity is considered. For this reason, phenological, physiological and morphological characteristics, called functional traits have been identified [6]. These determine how each species interacts with the ecosystem and how it reacts to environmental factors such as air pollution [7]. For example, trees with specific characteristics of leaf macro-morphology reduce air pollution by retaining atmospheric particulate matter (PM) that settles on the leaf surface [8]. These characteristics are not always considered in the selection of urban trees because the choice has mainly been focused on taxonomic or non-functional categorizations [9]. Moreover, there are invasive species that have a great ecological impact on the diversity of native species, affecting their ecosystem effects in urban areas and making it difficult to identify alterations caused solely by air pollution [10].

Functional traits, and in particular leaf morphology, are fundamental aspects in the choice of urban vegetation. It has been identified in different studies that leaf morphological traits act as precise monitors to determine the effects of anthropogenic actions on ecosystems [7,11]. These also serve to predict the synergies between the different ecosystem benefits provided by each plant species [6,12]. In addition, there are studies that report how leaf morphological traits have been used to evaluate invasive species that impact the ecological processes of other species [10,13]. Thus, analyzing the existing literature in relation to the leaf macro-morphological traits (LMTs) of trees and their capacity to retain air pollutants in urban areas may allow establishment of criteria and strategies to take advantage of their ecosystem benefits. The ability to retain PM on the leaves means that trees can also be used as biomonitors of the presence of certain urban air pollutants. Indeed, this literature review serves as a reference point for tree planning in the context of sustainable urban development [14].

Functional traits are defined as the set of phenological, morphological, and physiological characteristics that plants possess to ensure their survival success, provide insight into ecological dynamics, and responses to changes in the environment [15]. Leaf area (LA), specific leaf area (SLA), and leaf dry matter content (LDMC) are among the most evident leaf functional traits (macro-morphological), due to their easy measurement and sensitivity to environmental variations, [16,17]. There are other leaf functional traits (micro-morphological), such as stomatal density, leaf thickness, leaf type, and minimal photosynthetic unit, that have a great influence on ecosystem processes such as primary productivity, biogeochemical cycles, and organic matter decomposition [6,18]. Hence, there are multiple traits that allow plants to maintain balance and ecological adaptability and, in a special way, leaf macro-morphology is an important part of the ecosystem benefits that the plant species provide to urban environmental dynamics [6,19].

The deposition of PM and gas molecules on vegetation (diffusion, interception, impaction, and sedimentation) is usually described in terms of a one-dimensional vertical deposition over a homogeneous layer of vegetation [20]. The physics of the particle deposition phenomenon is usually described as an air stream passing through the surface of a single leaf rather than an entire forest [21]. The main variable describing this phenomenon is deposition velocity, which is significantly dependent on the air particle size [22]. In the case of the smallest PM deposited on the leaf surface (≤2.5 µm), the influence of the particle resuspension phenomenon induced by the wind action has also been reported [23]. This resuspension phenomenon has to be considered, otherwise, prediction overestimates are observed in the particle capture efficiency of tree leaves. Meteorology is also important for the particle deposition phenomenon on tree leaves. For example, Vong et al. [24] reported that the deposition velocity of air particles on the leaves depended on the atmospheric stability of the boundary layer, which, in turn, depended on wind speed, temperature gradient, and relative humidity. Moreover, a common subsystem of urban areas is the street canyon. The vegetation of street canyons produces different effects on the deposition velocity of air particles on the tree leaves [25].

The type and size of air pollutant affects the deposition on the tree leaves. Thus, variation in the different LMTs alters their effectiveness in the capture of the various air pollutants [26]. Differences in leaf size and complexity are significant predictors of the PM deposition [27]. For example, Weerakkody et al. [28] found several beneficial LMTs for the PM capture. These traits were as follows: small size, complex shape, and hairy or waxy surface. Viecco et al. [29] also reported that tree species with smaller leaves tended to be more effective than species with larger leaves, which was attributed to the greater perimeter/surface ratio of smaller leaves, in that leaf size was inversely correlated with the accumulation of PM between 1–10 μm. Barwise and Kumar [30] reported that complex, small, and needle-shaped leaves were the most effective for capturing PM. In addition, the type of tree cover on urban land affected air pollutant retention capacity. The influence of a plant barrier on polluted air movement is affected by its density or, conversely, by its porosity [31]. When trees are healthy, the density can be determined by leaf macro-morphology [26]. Abhijith et al. [32] showed that wide, high, and low-porosity tree cover reduced pollutant concentrations in direction of the wind. However, the above LMTs were relative to each other and were influenced by external factors (e.g., wind speed and distance to the pollution source). In terms of deposition, urban evergreen tree species are preferable to deciduous species [33]. However, evergreen tree species are more susceptible to stressors such as climate change compared to deciduous tree species [34].

The main objective of this paper is to study, through a literature review, the LMTs most used for the selection of urban trees as air pollution biomonitors. In this study, the most studied air pollutants worldwide were considered in the context of their association with urban tree leaves. This literature review is relevant because it is a tool for tree species selection options for monitoring, controlling, and improving urban air quality. The paper is structured as follows. Initially, we describe the materials and methods used for this systematic literature review to collect and analyze the selected information. Subsequently, we present results and discussion concerning associated air pollutants, the most used LMTs, and applications in the context of urban air pollution. Lastly, the main conclusions are presented.

## 2. Materials and Methods

### 2.1. Information Detection

In this study, a systematic literature review was conducted for documents published worldwide between 2010–2020 about tree LMTs in urban areas in an air pollution context. The LMTs were studied with respect to their use as biomonitors of urban air pollution. The databases used were Scopus, ScienceDirect, and Google Scholar.

### 2.2. Information Analysis

Information analysis consisted of four stages. Stage 1. Document detection was performed with the following keywords (detection universe): leaf morphology, urban trees, and air pollution. A total of 26,506 documents were detected in the databases considered. The Scopus database detected 31.0% of the documents (8181), ScienceDirect detected 6.0% (1525), and Google Scholar detected 63.0% (16,800). Stage 2: From the keywords used in Stage 1, additional document detection was performed in the context of the most studied air pollutants. This was based on the following additional keywords: atmospheric particulate matter (PM), ozone (O_3_), nitrogen dioxide (NO_2_), sulfur dioxide (SO_2_), carbon monoxide (CO), carbon dioxide (CO_2_), PM_2.5_, volatile organic compounds (VOCs), PM_10_, and total suspended particles (TSP). These air pollutants correspond to those most commonly reported in the literature concerning urban areas [35].

Stage 3: From the keywords used in Stage 1, additional detection of documents was carried out under the context of the LMTs related to the retention of urban air pollutants, considering as a reference the LMTs reported by Sgrigna et al. [11] and Grote et al. [36]. The additional keywords considered were the following: leaf area (LA), specific leaf area (SLA), leaf dry matter content (LDMC), and leaf surface (LS). We excluded functional traits such as crown size, plant area index, and canopy morphology, to focus specifically on leaf macro-morphology. At this stage, a citation frequency index was considered [37] in the selected databases, where the importance associated with each keyword (variable) was organized by means of quartiles (Q) (e.g., for PM_2.5_ in Scopus, 2335 documents were detected in Stage 2 within the 8181 documents detected in Stage 1, i.e., index = 2335/8181 = 0.285; see Table 1). This index had a variation between 0.00–1.00, where the last quartile (Q4 = 0.00–0.25) associated keywords with the lowest citation frequency. In other words, the keywords with the highest citation frequency were associated with the first quartile (Q1 = 0.75–1.00). In the context of urban tree LMTs, the use of the Q index allowed detection of the most important air pollutants worldwide, LMTs, and applications of these LMTs. This is assuming that the most significant variables of a phenomenon were those associated with a higher frequency of citation in scientific documents. Although this assumption was not necessarily true, it was used in this study as a research guideline. Lastly, the following inclusion criteria were considered for the documents detected: (i) documents focused on leaf macro-morphology and urban air pollution, and (ii) documents focused only on urban trees and no other types of plants (e.g., lichens or ornamental plants).

Stage 4: From the keywords used in Stage 1, documents associated with leaf macro-morphology applications in the context of monitoring, controlling, and improving urban air quality were detected using the following additional keywords: air quality management, green infrastructure, and urban tree management. The applications of leaf macro-morphology were established from the guidelines of Miedema et al. [7], Milanović et al. [10], and Maclvor et al. [38].

Considering all the stages of the literature analysis, the following inclusion criteria were considered for the documents detected in stages 2, 3, and 4: (i) Stage 2, air pollutant documents that were in quartiles Q1 and Q2; (ii) Stage 3, LMTs that due to their citation frequency were found in quartiles Q1 and Q4; (iii) Stage 4, applications of leaf macro-morphology that were in quartiles Q1 and Q2. In this literature review, a total of 96 documents were selected, i.e., 1.17% of the total documents initially detected by the Scopus database.

Finally, the following statistical tests were applied to the data series for each of the variables considered in this literature review: descriptive statistics (minimum, maximum, mean, median, and standard deviation), Shapiro-Wilk normality test (p-value < 0.05), and Spearman correlation coefficient (rs). All the above statistical analyzes were carried out using Ri386 3.6 software and with 95% confidence.

## 3. Results and Discussion

### 3.1. Urban Air Pollutants Associated with LMTs

**Figure 1:**
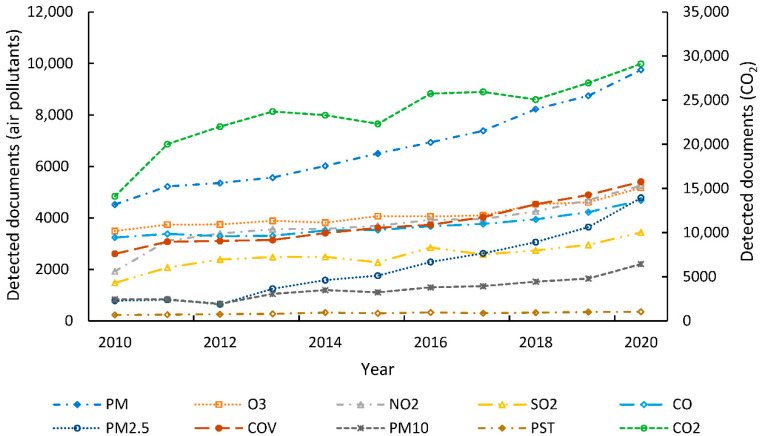
Temporal trend in the citation frequency of detected urban air pollutants by the Scopus database (documents considered, *n* = 8181).

The results show that the most studied air pollutants in the context of this literature review were the following (citation frequency, Q index): PM—all fractions between 1 and 100 um (Q1 = 0.760), O_3_ (Q2 = 0.586), PM_2.5_ (Q2 = 0.504), and PM_10_ (Q3 = 0.423) (Table 1). Other air pollutants showed a lower Q index: CO_2_ (Q3 = 0.427), CO (Q3 = 0.317), NO_2_ (Q3 = 0.295), and SO_2_ (Q3 = 0.287). The lower citation frequency of the latter group of air pollutants was possibly because the measures implemented for their control were more effective, or that the concentrations detected worldwide had a lower impact on public health in relation to the first group of air pollutants [35]. It was noted that, in recent years, CO_2_ has had greater interest in studies related to air pollution compared with other pollutants (Figure 1). This trend may be related to interest in global warming. Moreover, it was evidenced that the citation frequency of all pollutants has shown an increase in recent years. However, air pollutants such as VOCs and TSPs showed a low Q index (Q4 = 0.149 and Q4 = 0.034, respectively). Thus, these air pollutants were not considered in the discussion of results of this study. Guerreiro et al. [35] also excluded air pollutants with a lower citation frequency from their statistical analysis. Therefore, this study focused on air pollutants with a Q index within the first and second quartiles (i.e., PM—all fractions, O_3_, and PM_2.5_).

The findings showed, worldwide, for PM all fractions showed a higher citation frequency (Q = 0.760) under the context of urban tree leaves. For example, in China’s most industrialized cities, such as Beijing, Guangzhou, Nanjing, and Shanghai, this was the most cited air pollutant. This is probably due to high emissions from industrial activities, the use of domestic fuels, and heavy traffic [39]. According to Karagulian et al. [40], PM emissions increased worldwide, by 8.0–16.0%, during the period between 1990 and 2014. When comparing the documents analyzed, it was observed that the worldwide PM sources were similar. In this study, the order of importance of these PM sources was as follows: biomass burning (39.7%) > vehicular traffic (25.2%) > domestic fuels (20.1%) > industries (15.0%). Karagulian et al. [40], Tao et al. [41], and Yang et al. [42] reported similar results. Therefore, the measurement of PM concentration retained on the surface of urban tree leaves was considered as an effective monitor to determine air quality and human health status in different cities globally [40].

Additionally, the results showed that PM concentrations associated with studies on urban tree leaves in countries such as China and the United States [43,44], as well as the European continent, were elevated (Table 2). Ostoić et al. [45], Selmi et al. [46], and Reche et al. [47] reported similar findings. In these studies, the annual PM concentrations reported exceeded the maximum standards established by the World Health Organization (WHO) of 10 μg/m^3^ for PM_2.5_ and 20 μg/m^3^ for PM_10_ [48]. On average, in the documents analyzed, the United States exceeded the maximum standards for PM_2.5_ and PM_10_ by 64% and 364%, respectively. In Europe, these were values were 2140% and 147% of the maximum standards for PM_2.5_ and PM_10_, respectively. In China, PM_2.5_ and PM_10_ concentrations were exceeded by 6210% and 96%, respectively. During the study period, PM_2.5_ concentrations were regulated in China only until 2012. However, China accounted for 66.0% of the studies analyzed (e.g., [35,40,42,49]). This was possibly associated with the high concentrations of urban PM reported, which were related to emissions from large industrial sectors and high traffic density. In the context of urban tree leaves as biomonitors of air pollution, the results showed that the maximum concentrations reported in China during the study period for PM_2.5_, PM_10_, and O_3_ were 631, 136, and 268 μg/m^3^, respectively. Lastly, the findings indicate that studies on LMTs of urban trees as a biomonitors of air pollution were conducted at sites with high PM concentrations.

The results show that the average concentrations of air pollutants differed comparatively between countries according to the type of air pollutant analyzed in the context of the studies detected on LMTs of urban trees. For example, China was ranked first in air pollution, as reported concentrations of PM_2.5_, PM_10_, O_3_, and SO_2_ were 154.9, 92.0, 83.6, and 10.8 μg/m^3^, respectively (Table 2). However, for NO_2_ (14.4 μg/m^3^) and CO (0.74 μg/m^3^) the concentrations were in an intermediate range. These concentrations were reported in studies on urban tree leaves conducted in China’s largest and most industrialized cities (e.g., Beijing, Guangzhou, Nanjing, and Shanghai). Conversely, in European Union member countries such as Germany, Italy, France, Spain, and the Netherlands, NO_2_ concentrations ranked first (average = 43.7 μg/m^3^). This air pollutant is one of the main causes of soil acidification and eutrophication, and promotes PM and O_3_ formation [50]. Relative to the other air pollutants reported in Europe, the results showed that PM_2.5_, O_3_, and SO_2_ had comparatively intermediate concentrations of 114.5, 50.3, and 7.08 μg/m^3^, respectively. Lastly, in the USA the reported concentrations of PM_2.5_, O_3_, NO_2_, and SO_2_ were comparatively low at 13.9, 37.8, 0.430, and 0.52 μg/m^3^, respectively. Nevertheless, the highest CO concentrations were reported in the USA (41.1 µg/m^3^). This is possibly due to a lack of effectiveness in the control measures for human combustion sources in urban areas [40,51].

In relation to PM_2.5_, it was reported that this air pollutant usually had regional behavior, which implied that it could be transported from areas of high pollution to urban areas where annual concentrations did not exceed the standards allowed by WHO [39,52]. In addition, the studies showed that the high PM_2.5_ concentrations deposited on the tree leaves showed no significant seasonal differences, even though rainfall in winter tended to wash the deposition of this air pollutant [53]. An analysis with Spearman’s coefficient showed a very strong positive correlation (rs = 0.926; *p*-value < 0.001) between PM_2.5_ and O_3_ concentrations detected on the leaf surface (Table 3). Jia et al. [54] and Wang et al. [55] reported that under high O_3_ concentrations, and in the presence of atmospheric oxidation, the formation of secondary particles such as PM_2.5_ were promoted. Lastly, a considerable positive correlation (rs = 0.707; *p*-value = 0.049) was also observed between PM_10_ and PM_2.5_ concentrations detected on the leaf surface. Gómez-Moreno et al. [51], and Jia et al. [54] reported similar results.

The findings show that there were different PM sources, both natural and artificial, in the context of tree LMTs as a biomonitor of urban air pollution. According to Karagulian et al. [40], natural sources contributed most to elevated PM_10_ concentrations. In contrast, combustion sources had a greater influence on the formation of PM_2.5_. The results suggest that high PM concentrations detected on urban tree leaves can be explained by emissions from human activities, weather conditions, and the interaction of different compounds present in the atmosphere [51,56]. The results also show that PM and O_3_ had a significant influence on air quality in studies on urban tree leaves. The results show in order of importance, PM—all fractions between 1–100 µm (Q1 = 0.760), O_3_ (Q2 = 0.586), and PM_2.5_ (Q2 = 0.504), were the most frequently studied air pollutants, followed by PM_10_ (Q3 = 0.423), CO (Q3 = 0.317), NO_2_ (Q3 = 0.295), and SO_2_ (Q3 = 0.287). This trend was most evident in the countries identified as the most industrialized (e.g., China, the United States, and Germany). PM was also of great interest due to its serious direct effects on urban public health [41,42].

### 3.2. LMTs Associated with Air Pollution Biomonitoring

**Figure 2:**
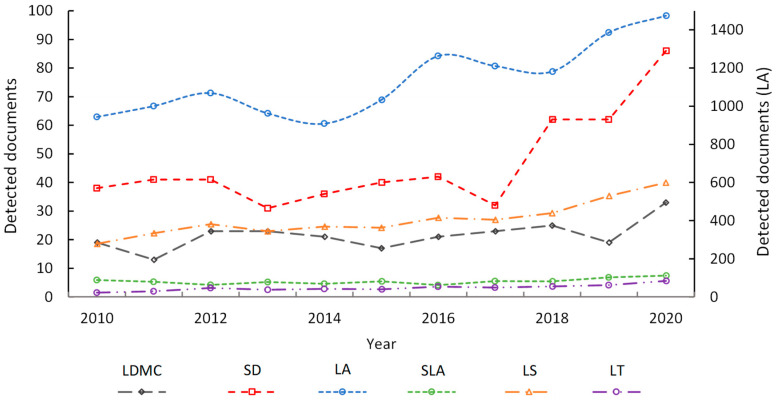
Temporal trend in citation frequency for LMTs detected in studies on urban air pollution from the Scopus database (*n* = 8181). LA = Leaf area, SLA = Specific leaf area, LDMC = Leaf dry matter content, LS = leaf surface, LT = Leaf thickness, and SD = Stomatal density.

The results show that in studies on leaf macro-morphology of trees and urban air pollution, the most used LMTs were LA and SLA (e.g., [6,57]). In relation to LA (Q = 1.000), SLA was Q4 (Q = 0.096). Indeed, SLA had a higher citation frequency index (Q) with respect to the other LMTs detected; for example, compared to LS (Q4 = 0.080) and LDMC (Q4 = 0.020) (Table 1). In this study, 64.0% of the documents detected reported LA and SLA (e.g., [58,59,60]). However, the use of these LMTs was also evidenced in studies on ecosystem benefits [1,6,61], determination of photosynthetic levels [62], and urban soil fertility [63]. It was also observed that in recent years the citation frequency for LA increased significantly compared to the other LMTs detected [64,65] (Figure 2). Lastly, the studies detected showed the use of other leaf traits with a very low citation frequency. In descending order, the Q index for these leaf traits in relation to LA was as follows: LS (Q4 = 0.080) > LDMC (Q4 = 0.020) > LT (Q4 = 0.013) > SD (Q4 = 0.001) (Table 4). These leaf traits were used less frequently in urban air pollution studies and were not considered in depth in this study.

The findings show the importance of LA as a trait that allows the study of different environmental factors, such as evapotranspiration, light interception, response to irrigation, and ecological factors such as photosynthetic efficiency and plant growth in urban green areas [14,62]. It was also reported that LA is commonly used in studies on urban air pollution due to its easy measurement and quantification [64,65]. Indeed, LA and SLA are related to the determination of different ecosystem benefits. According to Hanisch et al. [6] and Lopez-Iglesias [66], LA and SLA are multiservice traits because, in addition to being fundamental factors in air pollution decrease, they help in the study of biomass production, erosion control, soil fertility, and control of water levels [6,57]. According to Borowy and Swan [62], soil plays a fundamental role in the performance of plant functional traits, which is why both LA and SLA have a significant relationship with soil fertility. These leaf traits also make it possible to assess the response of plant species to their environment and are associated with carbon sequestration. The latter is a fundamental ecosystem service in the regulation of global warming, and is associated with the photosynthetic functions of the plants [17]. In addition, Kichenin et al. [67] reported that these leaf traits varied with the altitudinal gradient. It was shown that both LA and SLA increase as the altitude increases. This allows plant species that present these leaf traits in greater proportion to dominate other species in the ecosystem [7]. The results also hint that these leaf traits are influenced by altitudinal gradients and the meteorological and climatic conditions of a given region [68,69].

Additionally, Singh et al. [58] reported that LA in tree species is directly related to PM concentrations, because at higher LA, tree species retain more PM [70]. Some researchers [16,17] suggested that the previous trend was a response to urban pressures and that this varied with the tree species considered. Thus, the findings suggest that the identification of these variations in leaf traits may have a significant influence during the selection of tree species for the urban air pollution monitoring [1,58,65]. During the PM_2.5_ study, other leaf traits were also reported that allowed a better understanding of its retention by urban trees, including trichomes and stomatal density [14,71]. Previous studies have suggested the usefulness of these leaf traits in air quality analysis in the context of urban tree species. Indeed, leaf traits have become a tool for the study and management of air quality and ecosystem benefits provided by urban trees [72,73].

The results show that LMTs such as LA and SLA, and to a lesser extent LDMC, can be modified by invasive plant species. These species have leaf traits that allow them to dominate plant communities and functional structure in urban areas [60]. Hence, the functional diversity of native species decreased and was homogenized as invasive species increased [63]. In other words, the functioning and production of ecosystem benefits of native species was altered, which directly influenced the management of urban air quality [10,63]. The findings also suggested that both LA and SLA are influenced by urbanization and temperature, as these factors (anthropic and climatic) exert pressure on these LMTs. Urbanization has been associated with an increase in leaf area, due to soil conditions and the higher albedo observed in urban areas [74]. Pandey and Singh [64] demonstrated that LA and SLA increased under high humidity conditions, possibly as an adaptive response to climate change [75].

In relation to the order of importance in the use of LMTs in an air pollution context, a significant number of documents detected (≈80.0%) used LA to establish different environmental or ecological conditions, either of the same plant or of its ecosystem, possibly due to its ease of measurement. This trend was constant in the documents detected regardless of geographical location (China, Europe, United States, or Latin America), and was repeated for different tree species. Lastly, LA provided information about the different air pollutants retained on tree leaves (e.g., heavy metals, hydrocarbons, PM, CO, and O_3_), which supported their use in studies on urban air pollution [64].

### 3.3. LMT Applications

Our results suggest three main applications of leaf macro-morphology in the context of urban air pollution (*n* = 10): green infrastructure (50.0%) [10,17,38,67,76], air quality management (30.0%) [36,70,77], and tree management (20.0%) [7,48]. This trend was consistent across the documents detected, regardless of the study location. These applications showed different citation frequencies, but all were possibly related to each other by their benefits in increasing ecosystem benefits, such as improving air quality and climate regulation [78], and by their benefits on tree structure, which involved proper vegetation planning in urban spaces [79]. In relation to urban green infrastructure, the findings suggest that green areas, corridors, roofs, and walls decreased air pollution concentrations and improve the quality of life of urban communities [78,79,80]. Urban planning of this green infrastructure, i.e., the definition of tree size, density, space between them, and maintenance, was a fundamental aspect for the selection of tree species [26,81]. Indeed, species-specific leaf traits (e.g., LA and SLA) had to be considered with respect to PM retention, ensuring that this green infrastructure could significantly reduce air pollutant concentrations in each area [32,65,82]. Therefore, the results suggest that urban green infrastructure can be used as a biomonitor of air pollution levels, because it oxygenates the urban environment through photosynthesis, dilutes polluted air, and absorbs and retains pollutants from urban air.

According to Barwise and Kumar [30], the interaction between green infrastructure and air quality improvement was mainly socio-ecological, and was influenced by the selection of tree LMTs for the reduction of urban air pollutant concentrations. However, it was recommended that this selection not only consider the reduction of air pollution but also consider other ecological factors related to the biological and ecological diversity of the species used for this purpose. The failure to consider these additional ecological factors could lead to inadequate management of urban trees [30,32,83]. The findings suggested that during green infrastructure design, the urban infrastructure itself be considered for the selection and location of the selected trees. The selection, location, and design of green infrastructure should not only be based on aesthetic aspects or the survivability of species, but the increasing pressures of urbanization on these urban ecosystems must be considered [20,84] to justify that its implementation provides ecosystem benefits fundamental to the survival and good quality of life of urban communities [14,17,85]. Therefore, studies on LMTs and functional diversity of trees must be increased and deepened to complement these types of sustainable urban applications [5,38].

The results suggest that the best way to manage air quality in urban environments is to reduce air pollutant emissions [35,40]. However, continued population growth, expansion of urbanization, and consumerism make this difficult [86]. Thus, strategies have been developed, e.g., control of multiple pollutants, transition to renewable energies, use of electricity to replace sources of PM_2.5_ emissions, and the implementation of green infrastructure in urban areas. These make it possible to manage air pollution levels in a practical, efficient, and economical way [87]. Vieira et al. [78] reported that complex structures in urban green areas (i.e., combination of trees, shrubs, and herbaceous) were an excellent strategy to decrease air pollution; not only was planting of trees important for the retention of atmospheric pollutants and climate regulation, but the adaptation of these complex urban ecosystems had to be managed [88]. Indeed, these complex ecosystems had to interact with climatic, physical, soil, biological, and ecological elements to guarantee the provision of ecosystem benefits of urban trees [20,78].

Additionally, the findings suggest the importance of assessing interactions that occur between the diversity, composition, and structure of urban green areas [89,90]. Pearse et al. [91] established that it was very important to break with the homogenization of urban vegetation, because this decreased the number of beneficial effects resulting from this type of ecosystem. Janhäll [20] reported that air pollutant concentrations in each urban area depended on the emission source and the design of urban vegetation. The studies reported that low vegetation was more effective, as its proximity to the soil surface increased the probability of atmospheric pollutant retention without the influence of wind [92]. Strong wind increased PM_10_ retention by urban vegetation [93], but this was different with PM_2.5_, because strong wind reduced the retention capacity of PM_2.5_ by the urban vegetation, due to its resuspension and transport through the air masses [94]. This tended to increase respiratory diseases of inhabitants, but as a mitigation strategy it was proposed to increase the use of tree species with a higher LA, which increased the surface area of retention for urban PM_2.5_ [95,96].

## 4. Conclusions

The findings of this study on the LMTs most used for the selection of urban trees as biomonitors of air pollution allow us to make the following conclusions. In order of importance, the most frequently biomonitored air pollutants by the LMTs of urban trees are the following: PM—all fractions between 1 and 100 µm (Q1) > O_3_ (Q2) > PM_2.5_ (Q2) > PM_10_ (Q3). In in recent years, there has been a growing interest in CO_2_ the context of global warming. The results suggest that PM is the most effective air pollutant for studying and evaluating urban air quality in the context of the tree LMTs. This is most evident in urban areas with high concentrations of this pollutant (PM_10_ ≥ 69.9 µg/m^3^; PM_2.5_ ≥ 123 µg/m^3^). Specifically, PM_2.5_ is the most used fraction in these studies. Positive correlations between PM_2.5_ and PM_10_ (rs = 0.707), and significant correlations between PM_2.5_ and O_3_ (rs = 0.926) were observed in studies involving the LMTs of urban trees. The PM retained on tree leaves has also been used to study other types of urban air pollutants (e.g., heavy metals or hydrocarbons). The most used LMTs for urban air pollution biomonitoring are in order of importance LA (Q1) and SLA (Q4). These LMTs are frequently used because of their easy measurement and quantification. It is suggested that in urban areas, LA is directly related to the amount of PM retained on tree leaves. Unlike PM_10_, with PM_2.5_ it is necessary to consider the resuspension from the leaf surface of trees by the wind action.

Urban applications of LMTs suggest that green infrastructure is important for improving air quality by the capture of PM_2.5_, PM_10_, and O_3_, which are responsible for different diseases of public interest. Moreover, species should not only be selected for their ability to remove pollutants from the air, but must be chosen for their suitability to grow in urban environments, e.g., under specific climate conditions or considering invasive species. Lastly, the findings of this study are relevant to deepen knowledge regarding the use of tree LMTs to biomonitor air pollution in urban areas. This research is relevant to the design and implementation of strategies for surveillance, control, and improvement of air quality associated with urban trees (e.g., green infrastructure).

## Figures and Tables

**Table 1:** Order of importance in relation to LMTs, urban air pollutants, and applications detected in the literature review (Q index).

Stage	Keywords	Databases						Total Docs.	Average Index (Q)	Quartile	Quartile Variation
		Scopus		ScienceDirect		Google Scholar					
		Docs.	Index	Docs.	Index	Docs.	Index				
1	Leaf morphology, urban trees, and air pollution	8181	1.000	1525	1.000	16,800	1.000	26,506	-	-	-
2	PM—all fractions	4203	0.514	1141	0.748	17,100	1.018	22,444	0.760	Q1	Q2, Q1, Q1
	O_3_	3464	0.423	484	0.317	17,100	1.018	21,048	0.586	Q2	Q3, Q3, Q1
	PM_2.5_	2335	0.285	637	0.418	13,600	0.810	16,572	0.504	Q2	Q3, Q3, Q1
	CO_2_	994	0.122	218	0.143	17,100	1.018	18,312	0.427	Q3	Q4, Q4, Q1
	PM_10_	1447	0.177	404	0.265	13,900	0.827	15,751	0.423	Q3	Q4, Q1, Q1
	CO	1194	0.146	149	0.098	11,900	0.708	13,243	0.317	Q3	Q4, Q4, Q2
	NO_2_	1632	0.199	153	0.100	9810	0.584	11,595	0.295	Q3	Q4, Q4, Q2
	SO_2_	1379	0.169	157	0.103	9910	0.590	11,446	0.287	Q3	Q4, Q4, Q2
	COVs	1216	0.149	255	0.167	2180	0.130	3651	0.149	Q4	Q4, Q4, Q4
	TSP	42	0.005	37	0.024	1200	0.071	1279	0.034	Q4	Q4, Q4, Q4
3	LA	7457	1.000	2245	1.000	313,000	1.000	322,702	1.000	Q1	Q1, Q1, Q1
	SLA	1096	0.147	192	0.086	17,500	0.056	18,788	0.096	Q4	Q4, Q4, Q4
	LS	1030	0.138	835	0.372	35,300	0.113	37,165	0.080	Q4	Q4, Q3, Q4
	LDMC	196	0.026	41	0.018	4720	0.015	4957	0.020	Q4	Q4, Q4, Q4
4	Green infrastructure	750	1.000	345	1.000	19,400	1.000	20,495	1.000	Q1	Q1, Q1, Q1
	Air quality management	233	0.311	243	0.704	18,800	0.969	19,276	0.661	Q2	Q3, Q2, Q1
	Urban tree management	3	0.013	2	0.008	331	0.017	336	0.013	Q4	Q4, Q4, Q4

Note. Docs. = Detected documents. LA = Leaf area, SLA = Specific leaf area, LDMC = Leaf dry matter content, LS = Leaf surface.

**Table 2:** Global concentrations of air pollutants in studies on LMTs of urban trees (*n* = 25).

Site	Values	PM_2.5_	PM_10_	O_3_	NO_2_	SO_2_	CO
China	Maximum	631	136	268	23.6	19.3	1.26
	Minimum	34.0	42.0	18.0	6.50	4.30	0.48
	Mean	155	92.0	83.6	14.4	10.8	0.74
	Median	72.0	91.3	36.4	14.4	9.60	0.71
Europe	Maximum	224	173	120	87.1	12.9	0.78
	Minimum	10.0	10.1	1.02	0.31	1.18	0.02
	Mean	115	63.5	50.3	43.7	7.08	0.49
	Median	112	41.1	40.1	39.7	6.43	0.75
USA	Maximum	16.4	265	47.2	0.63	0.92	50.0
	Minimum	12.6	0.50	30.2	0.25	0.32	31.0
	Mean	13.9	70.4	37.8	0.43	0.52	41.1
	Median	13.4	0.42	37.5	0.41	0.46	41.5
Global	Maximum	631	265	268	87.1	19.3	50.7
	Minimum	10.0	0.50	1.04	0.25	0.24	0.28
	Mean	123	69.9	66.1	14.6	6.73	14.2
	Median	71.8	50.0	36.4	7.34	5.60	0.90

Note. Concentrations in µg/m^3^.

**Table 3:** Spearman correlation coefficients between air pollutants in studies on LMTs of urban trees (*n* = 25).

Air Pollutants	*p*-Valor	rs-Spearman
PM_2.5_–PM_10_	0.049	0.707
PM_2.5_–O_3_	<0.001	0.926
PM_2.5_–SO_2_	0.011	0.824
PM_2.5_–NO_2_	0.003	0.890
PM_10_–O_3_	0.039	0.731
PM_10_–SO_2_	0.089 *	0.636
PM_10_–NO_2_	0.017	0.798
O_3_–SO_2_	0.051 *	0.703
O_3_–NO_2_	0.022	0.780
SO_2_–NO_2_	<0.001	0.951

Note. * Non-significant correlations.

**Table 4:** Citation frequency analysis in relation to LA for leaf traits detected in studies on urban air pollution from the Scopus database (*n* = 8181).

Leaf Traits	Index (Q)	Citation Frequency
LA	1.000	80.0%
SLA	0.096	9.60%
LS	0.080	8.00%
LDMC	0.020	2.00%
LT	0.013	1.30%
SD	0.001	0.10%

Note. LA = Leaf area, SLA = Specific leaf area, LDMC = Leaf dry matter content, LS = leaf surface, LT = Leaf thickness, and SD = Stomatal density.

## Data Availability

Not applicable.

## Abbreviations

CO	Carbon monoxide
CO_2_	Carbon dioxide
LA	Leaf area
LDMC	Leaf dry matter content
LMTs	Leaf macro-morphological traits
LS	Leaf surface
LT	Leaf thickness
NO_2_	Nitrogen dioxide
O_3_	Ozone
PM	Atmospheric particulate matter
PM_10_	Atmospheric particulate matter ≤ 10 um
PM_2.5_	Atmospheric particulate matter ≤ 2.5 um
Q	Quartile
rs	Spearman coefficient
SD	Stomatal density
SLA	Specific leaf area
SO_2_	Sulfur dioxide
TSP	Total suspended particles
VOCs	Volatile organic compounds
WHO	World Health Organization
